# Impacts of plant and soil stoichiometry on species diversity in a desert ecosystem

**DOI:** 10.1093/aobpla/plac034

**Published:** 2022-08-10

**Authors:** Suwan Ji, Lamei Jiang, Dong Hu, Guanghui Lv

**Affiliations:** College of Ecology and Environment, Xinjiang University, Urumqi 830017, China; College of Ecology and Environment, Xinjiang University, Urumqi 830017, China; College of Life Science, Northwest University, Xi’an 710069, China; College of Ecology and Environment, Xinjiang University, Urumqi 830017, China

**Keywords:** Desert ecosystem, ecological stoichiometry, leaf, soil, species diversity

## Abstract

Plant and soil stoichiometric ratios can be used to explain changes in the structural and functional characteristics of plant communities. Exploring the relationships between the stoichiometric ratios and plant diversity is helpful to further elucidate the effects of soil and nutrient constraints on community vegetation. However, such studies remain poorly understood in desert ecosystems. In this study, we analysed the effects of soil moisture and salt content on soil and leaf stoichiometry, species diversity and their relationships in the desert ecosystem of the Ebinur Lake basin. The results showed that: (i) Compared with the low soil moisture and salinity (SW2) environment, the soil and leaf C, N, P contents and soil stoichiometric ratios were larger in the high soil moisture and salinity (SW1) environment, and the leaf stoichiometric ratios were smaller. (ii) In SW1 environment, species diversity was negatively correlated with soil C:N and C:P, but weakly correlated with soil stoichiometric ratios in SW2 environment. In addition, the relationships between it and leaf stoichiometric ratios were reversed in different moisture and salinity environments. (iii) Structural equation modelling showed that leaf C:P, C:N and soil C:P had strong effects on species diversity. This research aims to provide a scientific reference for maintaining plant diversity, vegetation reconstruction and ecosystem restoration in desert areas, and enrich the ecological stoichiometric theory of desert ecosystems.

## Teaser

Soil and plants in the desert ecosystem of the Ebinur Lake basin were mainly limited by N. The stoichiometric characteristics of the leaves and soil showed significant differences in the different moisture and salinity environments. The species diversity indices in high moisture and salinity were lower than those in a low moisture and salinity environment, which was related to soil salinity. The stoichiometric ratios of the leaf and soil were closely related to the species diversity, and the relationships between them were affected by soil moisture and salinity. Compared with soil, leaf stoichiometric ratios had a greater effect on species diversity.

## Introduction

Carbon (C), nitrogen (N) and phosphorus (P), as the most important biogenic elements of biological organisms, have significant impacts on the structure and functions of ecosystems (Westheimer 1987). C is the universal medium of all life forms on earth, and the interactions among many elements are regulated by the ratio of C to other nutrients (Melillo *et al.* 2003). As essential mineral nutrients for plant growth and common limiting elements in ecosystems, N and P have functional relationships in plants (Zhang *et al.* 2003), including N:P ratio in live vegetation is a diagnostic tool for identification of general N or P limitation, and they are major factors that regulate the decomposition rate of plant litter and the C balance of ecosystems (Güsewell and Freeman 2003). Soil C:N:P ratios are closely related to the decomposition rate of litter, the number of soil microorganisms and the long-term accumulation of soil organic C and nutrients (Dormaar *et al.* 1990; Näsholm *et al.* 1998). Tian *et al.* (2010) studied the pattern and changes of soil C:N:P ratios in China and found that changes in the soil C:N ratios were relatively small in different climatic zones, soil orders, soil depths and weathering stages, while the spatial heterogeneity of C:P ratios and N:P ratios was high and changed greatly. This is because there is a high correlation coefficient between C and N concentrations, while P content of soil is related to parent material P content and weathering stage, both of which are spatially heterogeneous (Tian *et al.* 2010).

At the individual plant level, the interactions among C, N and P and the relationships between the plant and its external environment together determine the nutrient level and growth and development process of the plant (Bazzaz and Grace 1998; Güsewell 2004). The stoichiometric ratios of C:N:P can also be used to analyse the C cycle and the balance and restriction of N and P elements in an ecosystem (Wang and Yu 2008). A large number of studies have shown that the leaf N:P critical ratio can be used as an indicator for judging the nutrient supply status of the environment for plant growth (Wassen *et al.* 1995; Aerts and Chapin 1999; Güsewell 2004). Based on their studies that the C:N:P ratio of an organism would exhibit a strong relationship with its growth rate, Sterner and Elser (2002) proposed the productivity hypothesis, which states that organisms with high productivity have lower C:P and N:P ratios. Han *et al.* (2005) studied the relationships between the leaf N:P ratios and latitudes of 753 terrestrial plants in China and found that the plant and soil stoichiometry exhibited wide heterogeneity among different communities and ecosystems. Other study has shown that the leaf nutrient content depends on the dynamic balance between soil nutrient supply and vegetation nutrient demand, and plant nutrient ratios often tend to be fixed (Wang and Yu 2008).

In previous studies, scholars mostly focused on the effects of biological factors, abiotic factors and human activities on the plant and soil stoichiometry (Elser *et al.* 2010; Zhang 2014; Schuster *et al.* 2016; Liu *et al.* 2020). In recent years, scholars have explored the relationships between plant diversity and the stoichiometric ratios of leaf or soil, mainly focusing on grassland and forest ecosystems. Chen *et al.* (2013) studied the subalpine meadow communities and found that the relationships between species diversity of high- and low-yield grasslands and plant C, N and P stoichiometric characteristics were significant and heterogeneous. Chen *et al.* (2020) found that soil C:N was closely related to species diversity in different coastal woodlands. In addition, Wang *et al.* (2018) studied the karst rocky desert ecosystem in south-western China and found significant correlations between plant diversity and soil ecological stoichiometry. Yang *et al.* (2018, 2019b) studied the relationships between species diversity of *Salix psammophila* community and *Artemisia ordosica* community and C:N:P of soil and vegetation in the sandy ecosystem of arid and semi-arid regions, and found that the species diversity of the two communities had positive correlations with soil C:N and N:P, while the vegetation N:P of the two communities had different effects on species diversity. By studying different ecosystems such as meadows, woodlands and desert, scholars found that there were significant differences in the relationships between ecological stoichiometry and the species diversity among different ecosystems and communities.

Terrestrial ecosystems are rich in a variety of C-containing chemicals that are difficult to decompose, and there are many factors that affect the C:N:P ratios, making terrestrial more complex than marine ecosystems (Zeng 2005). Therefore, research is still needed to determine the relationships between ecological stoichiometry and plant diversity, especially in desert ecosystems. The desert ecosystem is characterized by drought, little rain, large evaporation, serious wind erosion, low species diversity and productivity, which makes it very sensitive to human production activities. Moreover, its balance is easily broken and resilience is weak (Yang *et al.* 2019a). Therefore, by studying the relationships between the stoichiometric ratios of the leaf and soil and the species diversity, we can further reveal the relationships between vegetation and soil in desert ecosystems, as well as the life strategies of vegetation in arid areas. In this study, different soil moisture and salinity communities in the Ebinur Lake basin were used as the research objects to explore the maintenance mechanism of plant diversity in desert ecosystems. We hypothesize that both soil and plant stoichiometry affect species diversity but their influences may be different. To test the hypothesis, we ask the following research questions: (i) Are there differences in plant and soil stoichiometric characteristics in different soil moisture and salinity environments? (ii) How does leaf and soil stoichiometric ratios affect species diversity at different soil moisture and salinity levels? (iii) What are the key factors that affect species diversity in terms of the leaf and soil stoichiometric ratios? This study might provide reference for vegetation reconstruction and ecosystem restoration in desert areas, and also provide new methods and ideas for in-depth study of the response and adaptation mechanism of desert plants to soil environment.

## Materials and Methods

### Overview of study area

The study area is located in the Ebinur Lake Wetland National Nature Reserve in the north-west of Jinghe County in Xinjiang and the south-west of the Junggar Basin. It is the lowest depression and moisture and salinity collection centre on the south-western margin of the Junggar Basin. The climate in this area is dry, with low rainfall (100 mm per year, with uneven distribution), high wind and an evaporation capacity of 1315 mm, making this a typical temperate continental climate. The study area consists of grey desert soil, grey-brown desert soil and aeolian sandy soil. The desert ecosystem is affected by drought and salinity at the same time. The soil has high salinity and low moisture and nutrient availability. The average conductivity and pH values of the shallow (0–10 cm) soil are 5.41 ms cm^−1^ and 8.77, respectively, the average soil bulk density is about 1.38 g cm^−3^ and the average moisture content is about 7.19% (Zhang *et al.* 2016). The vegetation types in the desert area of the Ebinur Lake basin are mainly medium xerophyte and ultra-xerophyte desert plants (Zhang 2019).

### Sample plot set-up and quadrats investigation

**Figure 1.**
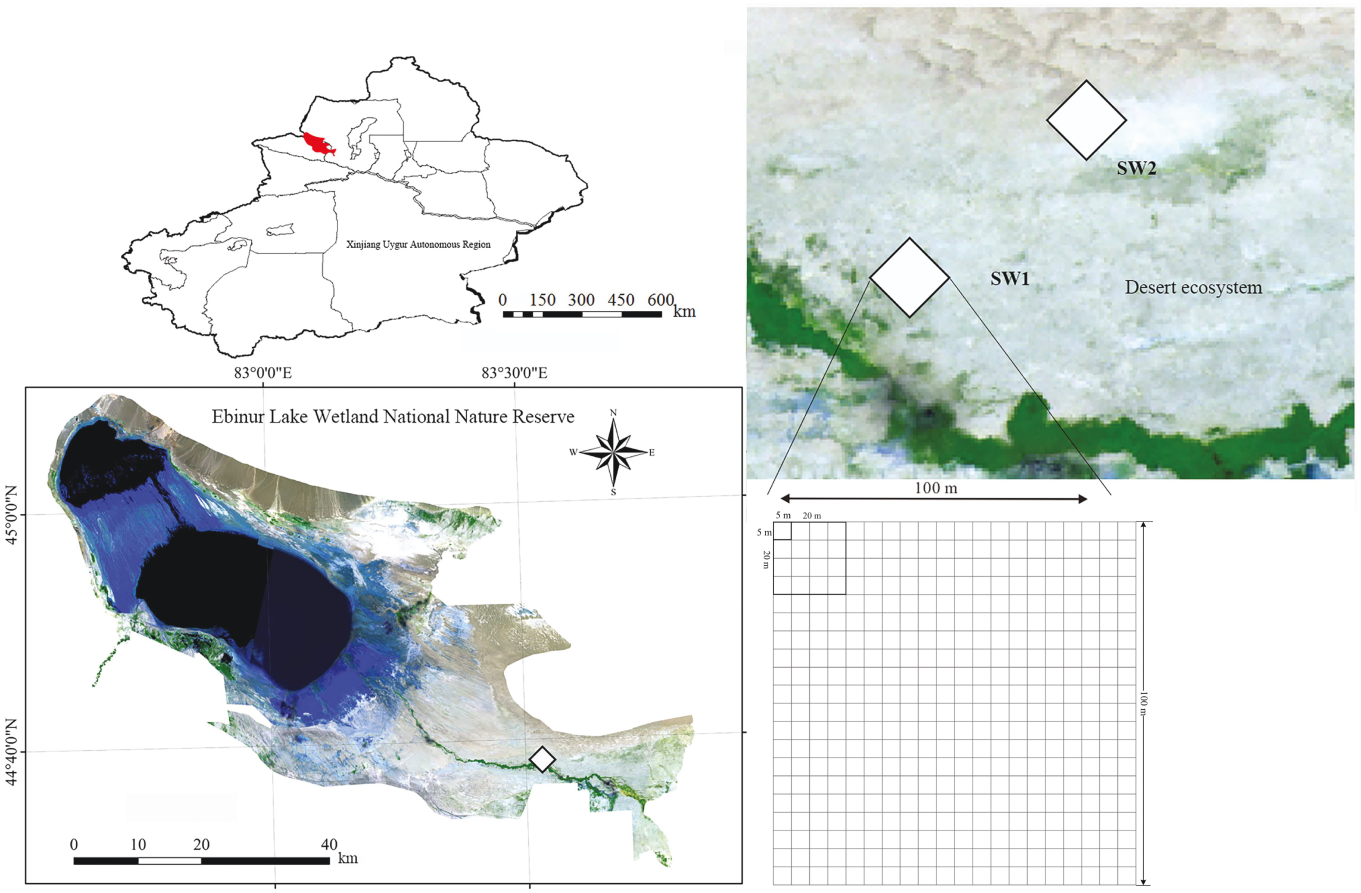
Location of two sample plots. SW1 is the high moisture and salinity plot, and SW2 is the low moisture and salinity plot.

Two 1-hm^2^ (100 m × 100 m) sample plots were laid out in the desert riparian forest on the northern bank of the Aqikesu River near the Dongdaqiao Maintenance Station in the reserve, located 300 m and 5000 m away from the riverbank, respectively. In this study, the community was studied at a scale of 20 m × 20 m, with 25 quadrats in each sample plot (Fig. 1).

In the survey sample quadrants, considering that soil C:N, C:P and N:P ratios in organic-rich topsoil could be a good indicator of soil nutrient status during soil development (Tian *et al.* 2010), the 0–20 cm soil layer was selected for sampling. First, soil was collected using an accurately weighed air aluminium box, and the fresh soil was weighed with a balance with an accuracy of 1/10 000. The soil was dried and then weighed again. These data were used to calculate the soil moisture content. Second, a soil sample was collected using a self-sealing bag, dried naturally and screened (100 mesh, except for the salinity) to determine the salinity (20 mesh), organic carbon (C), total nitrogen (N) and total phosphorus (P) contents **[see****Supporting Information—Table S1****]**Bao (2000).

It is known that in this study area, soils near the river had higher moisture and salinity than soils distant from the river (Zhang *et al.* 2018). The moisture and salinity of the two plots are presented in Table 1. The high moisture and salinity plot was termed SW1, and the low moisture and salinity plot was termed SW2. During the field investigation, the vegetation types in the two plots were recorded (Table 2).

**Table 1.** Soil moisture and salinity of two sample plots.

Sample plots	Moisture (%)	Maximum (%)	Minimum (%)	Salinity (g·kg^−1^)	Maximum (g·kg^−1^)	Minimum (g·kg^−1^)
SW1	12.873 ± 0.541^A^*	21.089	7.582	5.523 ± 0.331^A^	9.463	3.315
SW2	1.011 ± 0.047^B^	1.477	0.686	0.979 ± 0.04^B^	1.244	0.607

*Values are the mean ± SE, SE: standard error; different letters following values within a column indicate significant differences (*P* < 0.01). SW1: high moisture and salinity plot; SW2: low moisture and salinity plot.

**Table 2.** Vegetation survey list of the two sample plots.

	SW1			SW2		
	Species	Family	Biotype	Species	Family	Biotype
1	*Phragmites australis*	Poaceae	PG	*Phragmites australis*	Poaceae	PG
2	*Nitraria tangutorum*	Zygophyllaceae	S	*Nitraria tangutorum*	Zygophyllaceae	S
3	*Reaumuria songonica*	Tamaricaceae	SS	*Reaumuria songonica*	Tamaricaceae	SS
4	*Haloxylon ammodendron*	Chenopodiaceae	ST	*Haloxylon ammodendron*	Chenopodiaceae	ST
5	*Alhagi sparsifolia*	Fabaceae	SS	*Alhagi sparsifolia*	Fabaceae	SS
6	*Salsola collina*	Chenopodiaceae	AG	*Salsola collina*	Chenopodiaceae	AG
7	*Populus euphratica*	Salicaceae	T	Suaeda *glauca*	Chenopodiaceae	AG
8	*Apocynum venetum*	Apocynaceae	SS	*Seriphidium kaschgaricum*	Asteraceae	PG
9	*Halimodendron halodendron*	Fabaceae	S	*Horaninowia ulicina*	Chenopodiaceae	AG
10	*Lycium ruthenicum*	Solanaceae	S	*Calligonum mongolicum*	Polygonaceae	S
11	*Suaeda microphylla*	Chenopodiaceae	AG			
12	*Sonchus oleraceus*	Asteraceae	AG			
13	*Psathyrostachys juncea*	Poaceae	PG			
14	*Glycyrrhiza uralensis*	Fabaceae	PG			

SW1: high moisture and salinity plot; SW2: low moisture and salinity plot; AG: annual grass; PG: perennial grass; SS: subshrub; S: shrub; ST: small tree; T: tree.

The quadrats were investigated, and the abundance of all species in each quadrat was recorded. Healthy and mature leaves of each species from the four cardinal directions of individual plants were selected and samples weighing about 30 g were placed in large envelopes. The leaves were dried in the experimental plot (about at 30 °C for 1 day) and an oven (at 65 °C for 48 h), and were crushed and screened (100 mesh) for the determination of organic carbon (C), total nitrogen (N) and total phosphorus (P) contents **[see****Supporting Information—Table S1****]**. Based on the characteristic data of leaf C, N and P of each species in the quadrat, leaf C, N and P at community level took into account the abundance of the species calculating the average of trait values weighted by the relative abundance of each species, according to Garnier *et al.* (2004) as follows:


$$\begin{array}{l} CWM_{j}=\underset{i=1}{\overset{S}{\sum }}\;p_{ij}T_{ij} \end{array}$$ (1)

where CWM is the community-weighted mean value of a trait, *i* is the species in the quadrat (*i* = 1, 2,..., *S*), *j* is the community number, *p*_*ij*_ is the relative abundance of *i* in *j* and *T*_*ij*_ is the average trait value of *i* in *j*.

### Data processing and analysis

According to species type and abundance data within each quadrat, the Vegan package in R 3.6.1 (R Core Team 2019; Oksanen *et al.* 2020) was used to calculate the species diversity indices. CWM metrics were calculated by the FD package (Laliberté *et al.* 2014). The effects of moisture and salinity on the ecological stoichiometry of soil and leaf C, N and P and the species diversity indices were determined through variance analysis, and the relationships between the ecological stoichiometric ratios of the soil and leaf and species diversity were determined through regression analysis and the construction of a structural equation model. The structural equation model was constructed using AMOS 22.0 (IBM, USA), and the data were summarized, analysed and plotted in Excel 2010 (Microsoft, USA), R 3.6.1 and Visio 2007 (Microsoft, USA).

The selected species diversity indices are: (i) Margalef index (Wang *et al.* 2001), a commonly used richness index, is based on the relationships between the number of species and the total number of individuals in a community. (ii) Shannon index (Magurran 1998), which reflects the uncertainty degree of individual occurrence of species, is used to estimate diversity. (iii) Pielou index is the evenness index to judge the community uniformity, which is widely used in research practice (Ma *et al.* 1995). (iv) Simpson index (He *et al.* 1998), which reflects the richness and evenness of species in a community, is a comprehensive diversity index. The calculation method is referred to Zhang (2011).


$$\begin{array}{l} \begin{array}{ll} Margalef\;index & R=(S-1)/\ln N \end{array} \end{array}$$ (2)

$$\begin{array}{l} \begin{array}{ll} Shannon\;index & H=-\underset{i=1}{\overset{S}{\sum }}\;P_{i}\ln P_{i} \end{array} \end{array}$$ (3)

$$\begin{array}{l} \begin{array}{ll} Pielou\;index & E=H/\ln S \end{array} \end{array}$$ (4)

$$\begin{array}{l} \begin{array}{ll} Simpson\;index & D=\underset{i=1}{\overset{S}{\sum }}\;{\left(N_{i}/N\right)}^{2} \end{array} \end{array}$$ (5)

where *S* is the number of species, *N* is the abundance of all species, *N*_*i*_ is the abundance of species *i* and *P*_*i*_ is the proportion of species *i*.

## Results and Analysis

### Stoichiometric characteristics of soil and leaf C, N and P in different moisture and salinity environments

**Figure 2.**
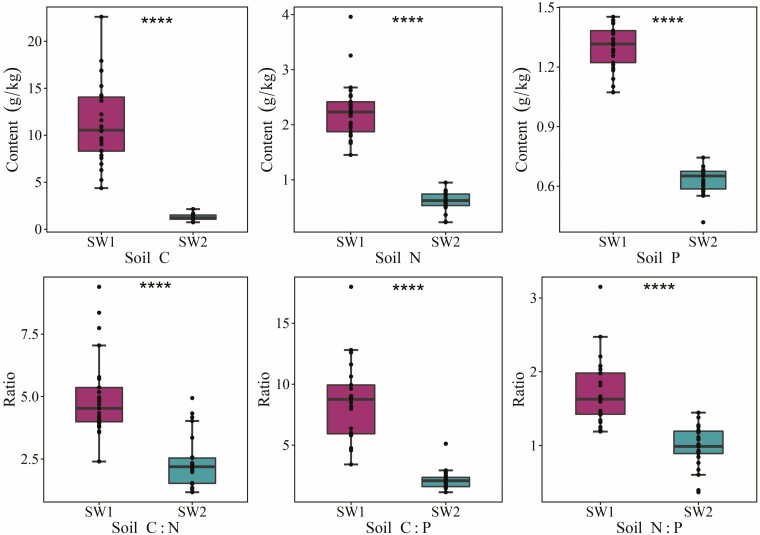
Characteristics of soil C, N and P stoichiometry in different moisture and salinity environments (*N* = 25). ****P < 0.0001. SW1 is the high moisture and salinity plot, and SW2 is the low moisture and salinity plot.

**Figure 3.**
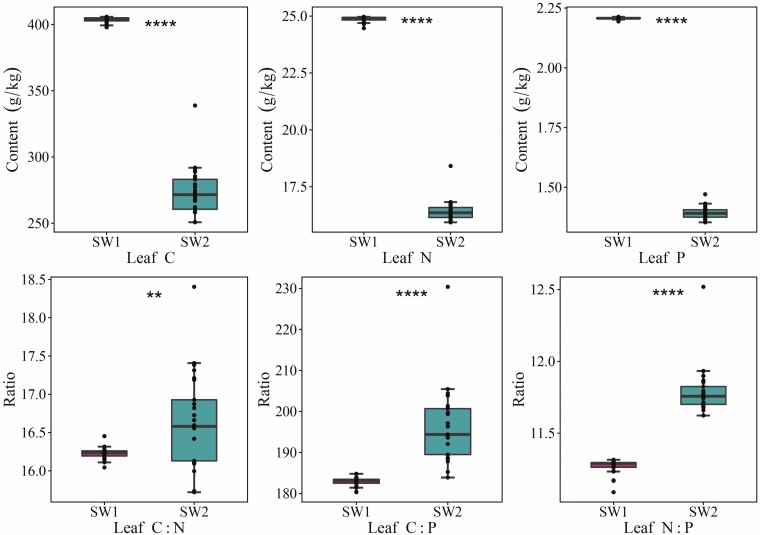
Characteristics of leaf C, N and P stoichiometry in different moisture and salinity environments (*N* = 25). ****P < 0.0001; **P < 0.01. SW1 is the high moisture and salinity plot, and SW2 is the low moisture and salinity plot.

There were significant differences in the contents and ratios of leaf and soil C, N and P in the different moisture and salinity environments (*P* < 0.01) (Figs 2 and 3). The contents and stoichiometric ratios of soil C, N and P in the SW1 were significantly higher than those in the SW2 environment (*P* < 0.01). In addition, the contents of leaf C, N and P in the SW1 were significantly higher than those in the SW2 environment (*P* < 0.01), while the leaf C:N, C:P and N:P ratios in the SW1 were significantly lower than those in the SW2 environment (*P* < 0.01).

### Characteristics of the species diversity of the desert communities

**Figure 4.**
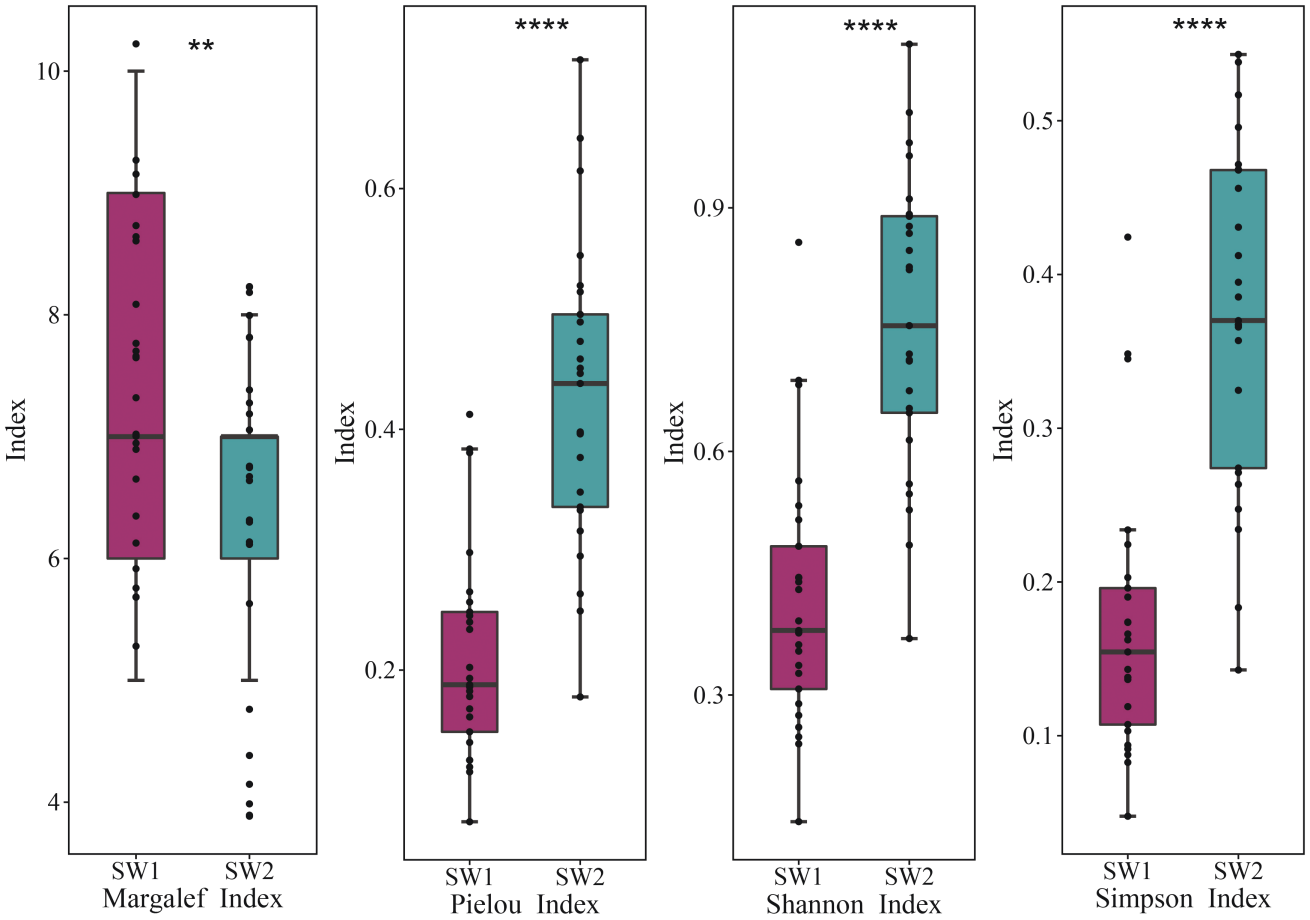
Characteristics of species diversity in different moisture and salinity environments (*N* = 25). ****P < 0.0001; **P < 0.01. SW1 is the high moisture and salinity plot, and SW2 is the low moisture and salinity plot.

The Margalef index (7.48) was significantly higher in the SW1 than in the SW2 environment (6.36), while the Pielou index, Shannon index and Simpson index values were significantly lower (*P* < 0.01) (Fig. 4). In this desert ecosystem, there were strong positive correlations among the Pielou index, Shannon index and Simpson index (*r* > 0.9); significant negative correlations between the Margalef index and Pielou index and between the Shannon index and Simpson index **[see****Supporting Information—Fig. S1****]**.

### Relationships between the species diversity indices and stoichiometric ratios of the soil and leaf in different moisture and salinity environments

**Figure 5.**
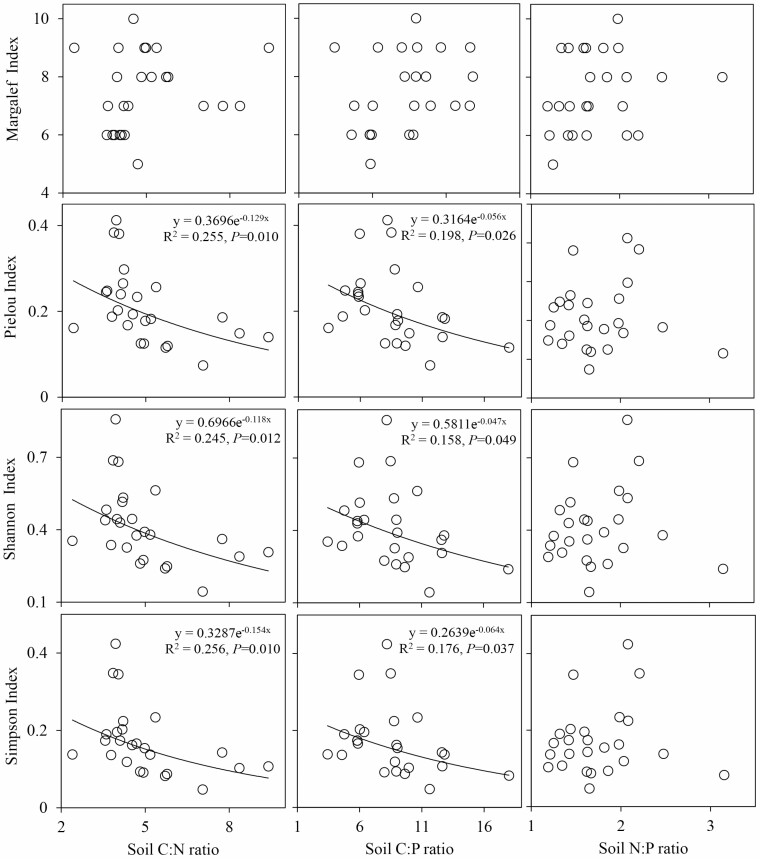
Relationships between the soil stoichiometric ratios and species diversity in SW1. SW1 is the high moisture and salinity plot.

**Figure 6.**
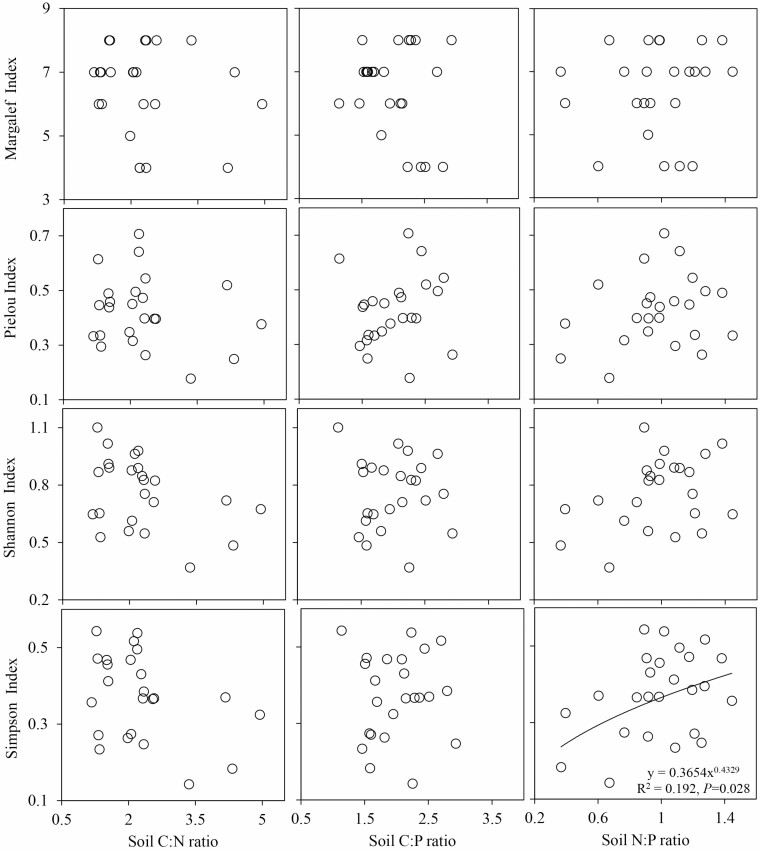
Relationships between the soil stoichiometric ratios and species diversity in SW2. SW2 is the low moisture and salinity plot.

The relationships between the species diversity indices and the soil C:N, C:P, and N:P ratios in the SW1 were different from those in the SW2 environment (Fig. 5). The soil C:N and C:P ratios had significant negative relationships with the Pielou index, Shannon index and Simpson index in the SW1 environment (*P* < 0.05). The relationships between the soil C:N ratio and the three species diversity indices were relatively strong, with coefficients of determination (*R*^2^) of 0.255, 0.245 and 0.256 for the Pielou index, Shannon index and Simpson index, respectively. The relationships between the plant species diversity and soil stoichiometric ratios were weak in the SW2 environment. Only the Simpson index increased with the increase in the soil N:P ratio, and the relationship between them was weak, with coefficients of determination (*R*^2^) of 0.17 (Fig. 6).

**Figure 7.**
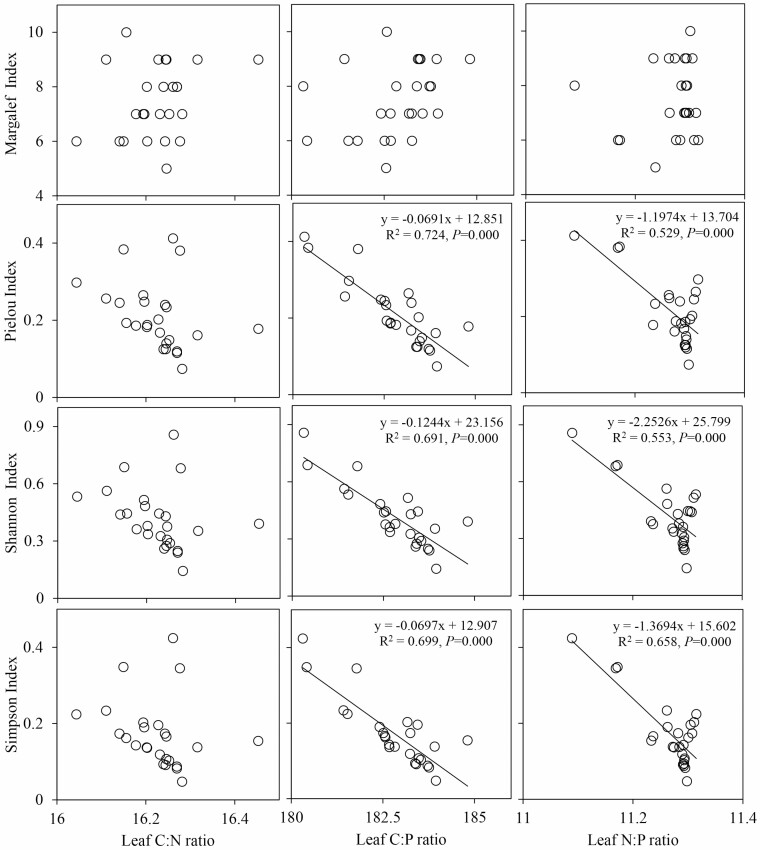
Relationships between the leaf stoichiometric ratios and species diversity in SW1. SW1 is the high moisture and salinity plot.

**Figure 8.**
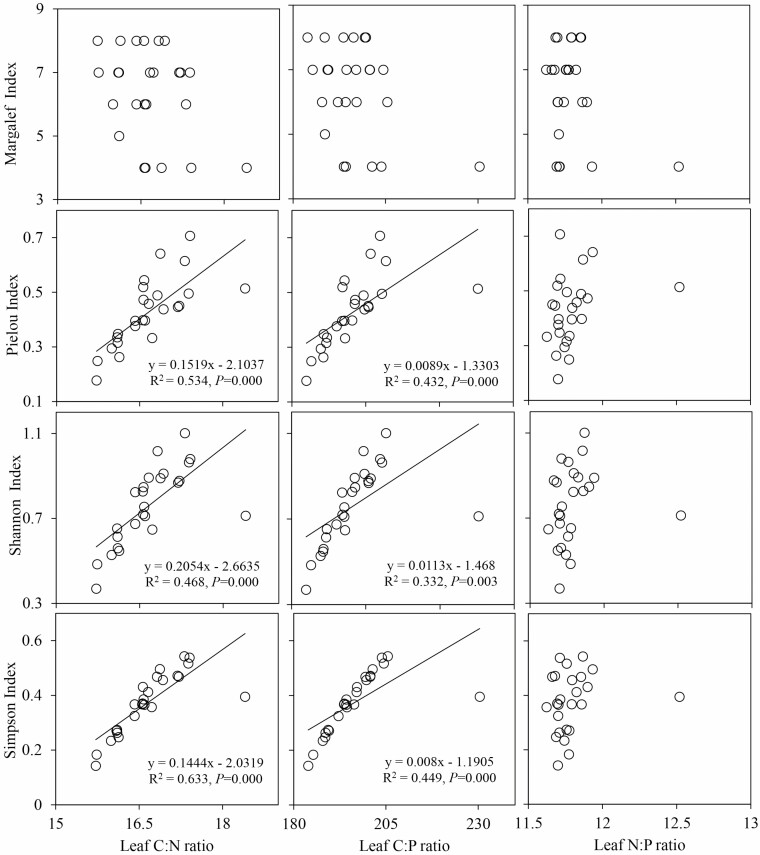
Relationships between the leaf stoichiometric ratios and species diversity in SW2. SW2 is the low moisture and salinity plot.

In the regression relationships between the species diversity indices and the leaf C:N, C:P and N:P ratios in the SW1 environment (Fig. 7), the Pielou index, Shannon index and Simpson index had extremely significant negative relationships with the leaf C:P and N:P ratios (*P* < 0.01), while the relationships among the other indicators were not significant (*P* > 0.05). In the regression relationships between the species diversity indices and the leaf C:N, C:P and N:P ratios in the SW2 environment (Fig. 8), the regression relationships between the Pielou index, Shannon index and Simpson index and the leaf C:N and C:P ratios showed extremely significant positive relationships (*P* < 0.01), while the other relationships were not significant (*P* > 0.05). In the significant regression relationships, the *R*^2^ ranged from 0.52 to 0.72 in the SW1 environment and from 0.33 to 0.63 in the SW2 environment.

### Effects of the soil and leaf C, N and P stoichiometric ratios on species diversity

The regression analysis of the species diversity indices and soil and leaf stoichiometric ratios revealed significant regression relationships between the species diversity and the ecological stoichiometric ratios in different moisture and salinity environments (Figs 5–8). In order to further analyse the internal relationships between desert vegetation and nutrients in soil and leaves, a structural equation model (SEM) was constructed based on the species diversity indices and stoichiometric ratios of the soil and leaf. Due to the internal correlation of species diversity index, the results of the SEM contain more redundant information **[see****Supporting Information—Fig. S1****]**. Therefore, we performed principal component analysis (PCA) on Margalef index, Pielou index, Shannon index and Simpson index. The results showed that *PC1* could account for 80.26% of variance (Table 3). According to the relationships between the four diversity indexes and *PC1* (see Formula (6)), four diversity indices were combined into a comprehensive diversity index, which was named species diversity and used as the dependent variable while other variables as independent variables in SEM.

**Table 3.** Total variance explained.

Component	Initial eigenvalues			Extraction sums of squared loadings		
	Total	% of variance	Cumulative%	Total	% of variance	Cumulative%
1	3.211	80.264	80.264	3.211	80.264	80.264
2	0.758	18.961	99.225			
3	0.019	0.486	99.711			
4	0.012	0.289	100			

The formula is as follows:


$$\begin{array}{ll} PC1= & -0.340\;Margalef\;index+0.554\;Pielou\;index\\ & +0.531\;Shannon\;index+0.543\;Simpson\;index \end{array}$$ (6)

**Figure 9.**
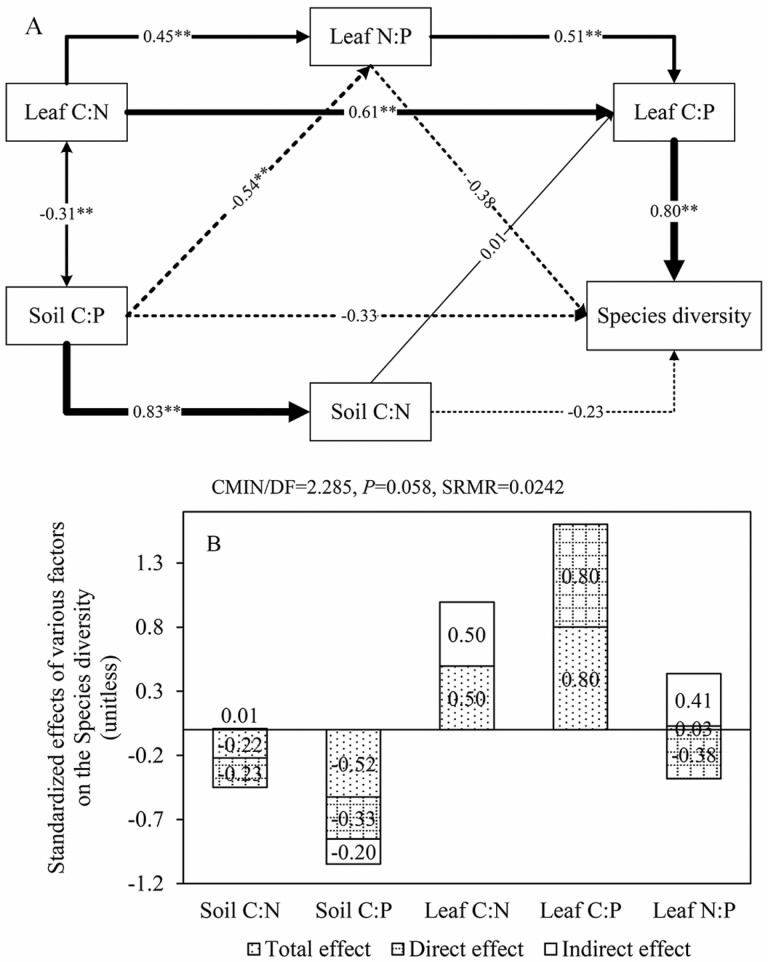
Structural equation model based on the effect of the soil and leaf stoichiometric ratios on species diversity. In (A), a single arrow indicates the assumed causal direction, and black real and imaginary lines indicate positive and negative relationships, respectively. The width of the arrow is in direct proportion to the intensity. The numbers near the arrow are normalized path coefficients, reflecting the magnitude of the causal relationship. The significance levels are expressed as follows: **P < 0.01. (B) Shows the normalized total effect of the corresponding structural equation model. The numbers are the values of total effect, direct effect and indirect effect. The indirect effect of each path is equal to the product of the coefficients of the paths it contains; indirect effect is the sum of the indirect effect of each path. Total effect = Direct effect + Indirect effect.

The fitting criteria of the structural equation model were CMIN/DF < 3, *P* > 0.05 and SRMR < 0.05. The model parameters used in this paper were CMIN/DF = 2.285, *P* = 0.058 and SRMR = 0.0242 (Fig. 9A). The constructed structural equation model showed that only leaf C:P had a significant direct effect on species diversity among the stoichiometric ratios, with an effect value of 0.80. Leaf C:N indirectly affected species diversity by affecting the leaf C:P (0.61) and N:P (0.45), with a total effect value of 0.50 (Fig. 9B). Leaf N:P had a significant positive effect on leaf C:P (0.51), and a negative direct effect (−0.38) and a positive indirect effect (0.41) on species diversity. Soil C:P showed a negative effect (−0.54) on leaf N:P, a positive effect (0.83) on soil C:N and its total effect on species diversity through direct and indirect effects reached −0.52.

## Discussion

### Stoichiometric characteristics of the soil and leaves in desert communities in different moisture and salinity environments

In desert ecosystems, ecological processes are centred on water resources (Castellanos *et al.* 2018). The lack of soil moisture can cause the N and P in arid areas to be in a state of scarcity at the same time by limiting the role of soil nutrients (He *et al.* 2008; Guo *et al.* 2016). Meanwhile, soil salinity is also considered to be a major environmental pressure in arid and semi-arid regions, and is highly correlated with moisture content (Zhang *et al.* 2018; Yang *et al.* 2021). The contents of soil organic C and total N in the research area were higher in the SW1 than in the SW2 environment (Fig. 2); this was consistent with the finding that soil moisture content was positively correlated with soil C, N and P (Wang *et al.* 2005). Besides, the wetter conditions benefit decomposition of leaf litter (Austin *et al.* 2004), and result in relatively higher nutrition levels in the areas nearer to the riverbank (Zhang *et al.* 2018). In this study area, compared with other soil properties, soil salinity has the greatest effect on soil microbial composition and diversity (Yang *et al.* 2021), which is manifested as inhibition of soil microbial activity and negative correlation with microbial biomass, which will lead to the reduction of soil organic matter decomposition rate (Yuan *et al.* 2007). Therefore, we guessed that the decrease of soil microbial activity led to slower decomposition of organic matter and more nutrient accumulation in the SW2 environment (Zhang *et al.* 2021). However, this study did not consider these factors, and extensive research is needed.

The soil C:N ratio is considered an important indicator for understanding soil organic matter composition and nutrient availability, and can also be used as a reference standard for determining the source of soil organic matter, the decomposition degree of soil organic matter and potential soil fertility (Swift *et al.* 1979; Paul 2007). In this study area, the mean soil C:N values of the two plots (SW1: 5.0, and SW2: 2.4) were much lower than those reported at the global level (Post *et al.* 1985) and in China (Tian *et al.* 2010) (13.3 and 14.4, respectively) (Fig. 2), which might be due to the low soil C and N contents in the desert ecosystem, leading to the low soil C:N. According to the inversely proportional relationship between soil C:N and the decomposition rate of soil organic matter (Dise *et al.* 1998), it was speculated that the decomposition and mineralization rates of soil organic matter in the SW1 were lower than those in the SW2 environment. Alternatively, soil microbial activity may have been inhibited in the high saline–alkaline soil environment (Yang *et al.* 2021). The soil C:P ratio in the SW1 was significantly higher than that in the SW2 environment. According to the higher availability of soil P represented by lower soil C:P (Wang and Yu 2008), it was determined that the availability of soil P in the SW2 environment was higher, which will help plants adapt to arid and barren environments. The soil N:P ratios of the two plots in this study area were significantly lower than the global value (Cleveland and Liptzin 2007) and Chinese (Tian *et al.* 2010) (0–10 cm) means (13.1 and 9.3, respectively). This is because the arid climatic conditions in temperate deserts result in low plant productivity, low soil C and N contents, low P leaching loss and a high soil P content, which lead to low soil C:P and N:P ratios (Tian *et al.* 2010). The low soil N:P ratio in the study area indicated that the limiting effect of N on soil was greater than that of P, especially in the SW2 environment.

Soil nutrients can change plant stoichiometry by affecting the absorption and utilization of nutrients by plants (Guo *et al.* 2016), and with a reduction in soil moisture, plants reduce their absorption of N and P (Cramer *et al.* 2009; Waraich *et al.* 2011; Sardans and Peñuelas 2012). In this study, the contents of leaf C, N and P were greater in the SW1 than in the SW2 environment, the same as soil C, N and P (Fig. 3). The leaf C:N, C:P and N:P ratios in the SW1 were lower than those in the SW2 environment, indicating that plants in SW2 environment had higher utilization efficiency of N and P elements (Schindler *et al.* 2003). This is consistent with the findings of Sardans (2012) and He and Dijkstra (2014), who proposed that their results were due to the improvement in the nutrient utilization efficiency or drought resistance, and that drought could increase the C:N and C:P ratios in the ecosystem and showed a positive effect on the plant N:P ratio and a negative effect on plant N and P. The lower leaf stoichiometric ratios in the SW1 environment reflected a faster plant growth rate (Elser *et al.* 2000, 2003). This is related to higher concentrations of leaf N and P, which ensure rapid plant growth (Zhang *et al.* 2021). Changes in the leaf C:N and C:P ratios in the different moisture and salinity environments were opposite to those of the leaf C, N and P contents, indicating that the leaf C:N and C:P ratios were dominated by the leaf N and P content, respectively (Niu *et al.* 2013). In the same way, the leaf N:P ratio was mainly determined by the leaf P content. Koerselman and Meuleman (1996) proposed in their study of different wetland systems in Europe that when the leaf N:P ratio was <14, it could be deduced that the plant was N-restricted. In the present study, the leaf N:P ratios in the high and low moisture and salinity environments were less than 14 (SW1: 11.3, and SW2: 11.8), indicating that the plants in the study area were mainly restricted by N, which was related to the fact that the soil was mainly restricted by N. To sum up, comparing the stoichiometric characteristics of soil and leaves in different moisture and salinity environments will help to reveal the nutrient utilization status of plants in desert ecosystems, and explore the adaptation mechanism of desert plants to the environment from different angles.

### Species diversity characteristics of desert plants in different moisture and salinity environments

As shown in Fig. 4, the species diversity indices in the study area were relatively low, indicating that the plant communities in the study area had a simple species structure and low community stability and were relatively sensitive to external disturbance (Wang *et al.* 2018). For species diversity, it is generally considered to increase with an increase in the moisture content (Zhang *et al.* 2021), while soil moisture and salinity play a combined role in this study area (Gong *et al.* 2018), and salinity has the greatest effect on plant diversity compared to other soil properties (Yang *et al.* 2021). The results showed that the species diversity indices in the SW1 were generally lower than those in the SW2 environment, which made us think that low salinity was thought to increase plant diversity in SW2 environment by improving seed germination rate and seedling survival (Li *et al.* 2004). Yang *et al.* (2021) also found that plant diversity in high moisture and high salinity was lower than that in low-moisture and low-salinity environment, suggesting that plants in high-moisture environment were suffering from salinity stress. The Margalef index was higher in the SW1 environment, and it was considered that when the soil salinity increased, new plants with a higher salinity tolerance appeared in the SW1 environment (Ma *et al.* 2015), such as *Populus euphratica*, *Lycium ruthenicum*, *Glycyrrhiza uralensis* and *Suaeda microphylla* (Table 2). The Pielou index, Shannon index and Simpson index increased significantly with the decrease in soil moisture and salinity, indicating that vegetation evenness and species dispersion were higher in the SW2 environment, while the species distribution was more concentrated and the species evenness was lower in the SW1 environment, similar to the result of Ma *et al.* (2012), who also believed that the soil salinity was the key factor affecting the composition and distribution characteristics of plant communities. Therefore, it was concluded that salinity stress might be the main reason for the significant difference in the species richness index (Margalef index) between the two habitats in the present study.

### Relationships between the stoichiometric ratios of the soil and leaf and species diversity

The relationships between the species diversity indices and the soil C:N, C:P and N:P ratios were greater in the SW1 than in the SW2 environment (Figs 5 and 6), which might be related to the small changes in the species diversity and soil stoichiometric ratios in the SW2 environment. In addition, plants in the SW2 environment grew slowly and had low nutrient concentrations and high N:P ratios, resulting in relatively stable soil environments, and high species and community stability (Li *et al.* 2000), so the relationships in the SW2 environment were weak. In this study, we found that the Pielou index, Shannon index and Simpson index decreased with the increase of soil C:N and C:P in the SW1 environment, indicating that the increase of the decomposition rate of soil organic matter and soil P availability could promote the species diversity of high moisture and salinity communities (Dise *et al.* 1998; Wang and Yu 2008). This is related to the low decomposition rate of soil organic matter, the low availability of soil P and the fact that plants in this community are stressed by salinity, which not only increases the nutritional requirements of plants, but also seriously interferes with the absorption of nutrients (Zhang *et al.* 2018). Von Liebig’s (1842) law of the minimum and Shelford’s (1913) law of tolerance state that the growth and development of plants depend on a smaller or larger amount of essential substances in the growth environment. Therefore, it was considered that the weak relationships between species diversity and soil stoichiometric ratios in SW2 environment were due to the fact that plants were mainly limited by moisture. He and Dijkstra (2014) also reported that the growth of plants under long-term drought conditions was mainly limited by moisture supply. The relationships between soil N:P and species diversity were weak in high or low moisture and salinity environments. Among them, soil N:P only had a significant effect on Simpson index in SW2 environment, which may be because both soil N:P and Simpson index increased with the increase of soil N content. This is in agreement with the result of Yang *et al.* (2019b), they believed that with the gradual development of the desert plant community, plants need more N and P for growth, and the community increased the consumption of soil N and P, then plant diversity will have an impact on soil N:P. A previous study in the desert ecosystem of Ebinur Lake basin found that the communities in this area were in an unstable state, and that vegetation tended to be limited by N and P along with the succession process (Zhang 2014). By studying forest ecosystems at different succession stages, scholars found that the N:P of each soil layer increased with the progress of succession (Liu *et al.* 2010), and that the C, N and P contents of the soil decreased significantly due to vegetation degradation (Yan 2007). Therefore, in the future research, by comparing and analysing the relationships between C:N:P ratio of soil and leaf and plant diversity in different succession stages of desert ecosystems, it is hope that the C:N:P ratio can be used to predict the possible changes of species composition and ecosystem functions in desert ecosystems over time.

The relationships between leaf stoichiometry and species diversity were significantly different in different moisture and salinity environments. There were negative correlations between species diversity and leaf C:P and N:P in SW1 environment (Fig. 7), indicating that the increase of species diversity could increase the leaf P content, which was related to the low available P in soil and the need for higher P concentration for rapid plant growth. However, there were positive correlations between species diversity and leaf C:N and C:P in SW2 environment (Fig. 8), which was related to the strategy used by desert community plants in which plants to adapt to arid habitats by reducing leaf N and P concentrations (Gong 2019). That is to say, with the reduction in leaf N and P concentrations, the leaf C:N and C:P ratios increased, resulting in positive correlations between them and species diversity. In summary, in different soil moisture and salinity environments, soil and leaf stoichiometric ratios had different effects on species diversity, indicating that fully considering the role of soil moisture and salinity can promote a more comprehensive understanding of the relationships between soil and leaf stoichiometry and species diversity. Compared with soil, the relationships between leaf stoichiometric ratios and species diversity were more complex. Hence, further studies on the relationships between species diversity and leaf stoichiometric ratios are needed.

The regression analysis results of the stoichiometric ratios in soil and leaf and species diversity demonstrated that the stoichiometric ratios of the leaf and soil were closely related to species diversity, which was also reflected in the results of the structural equation model (Fig. 9). The model showed that the effects of different stoichiometric ratios on species diversity varied. In the desert ecosystem of this study, species diversity increased with the increase of leaf C:P and C:N, and the relationship between species diversity and leaf N:P was complicated. Among them, leaf C:P had the greatest effect on species diversity, which was related to the important role of plant P content. In arid and semi-arid regions, P is involved in osmoregulation and the synthesis of soluble proteins, soluble sugars and proline, which help plants resist adversity and survive. Therefore, for species diversity, the plant P had a greater effect than the plant N content (Yang *et al.* 2018). In addition, species diversity decreased with the increase of soil C:P and C:N, indicating that the addition of soil total C and N would have a positive impact on species diversity. Overall, the effect of leaf on species diversity was stronger than that of soil stoichiometric ratios. Leaf C:P can be used as an important index to evaluate the response of species diversity of desert ecosystem in Ebinur Lake basin to ecological stoichiometry.

## Conclusions

Through analysis of the stoichiometric characteristics of the leaves and soil as well as the species diversity in different moisture and salinity environments, we found that soil and plants in this study area were mainly limited by N. The stoichiometric characteristics of the leaves and soil showed significant differences in the different moisture and salinity environments. The species diversity indices in high moisture and salinity were lower than those in low moisture and salinity environment, which was related to soil salinity. The stoichiometric ratios of the leaf and soil were closely related to the species diversity, and the relationships between them were affected by soil moisture and salinity. Compared with soil, leaf stoichiometric ratios had a greater effect on species diversity. According to other scholars’ studies on the impact of species diversity on stoichiometry, we will explore this issue in combination with soil microorganisms in later research.

## Supporting Information

The following additional information is available in the online version of this article—

**Table S1.** Experimental methods.

**Figure S1.** Correlation analysis of the species diversity index in the desert ecosystem.

plac034_suppl_Supplementary_Material

## Data Availability

Data from this study are available and can be accessed at the public data repository Dryad. https://doi.org/10.5061/dryad.gb5mkkwsg.

## Notes

**Plants, Ecosystems & Climate. Chief Editor**: Mary Heskel
