# Supplementary material for: Impacts of plant and soil stoichiometry on species diversity in a desert ecosystem
Source: AoB Plants. 2022 Aug 10;14(4):plac034. doi: 10.1093/aobpla/plac034 (PMC9422082; doi:10.1093/aobpla/plac034)
Supplement: plac034_suppl_Supplementary_Material [file plac034_suppl_supplementary_material.pdf]

Supporting Information:

Table S1. Experimental methods (Bao, 2000).

Classification	Index	Experimental method	Instrument
Soil	moisture content	drying and weighing method	1/10,000 electronic balance; Oven
	salinity content	conductivity method	Shaker; Conductivity meter
	organic carbon	potassium dichromate dilution thermal method	——
	total nitrogen	Kjeldahl method (H_2SO_4-mixed accelerator digestion)	Spectrophotometer
	total phosphorus	molybdenum-antimony anticolorimetry method ($HClO_4$-H_2SO_4 digestion)	Spectrophotometer
Leaves	organic carbon	potassium dichromate dilution thermal method	——
	total nitrogen	Kjeldahl method (H_2SO_4-H_2O_2 digestion)	Spectrophotometer
	total phosphorus	molybdenum-antimony anticolorimetry method (H_2SO_4-H_2O_2 digestion)	Spectrophotometer

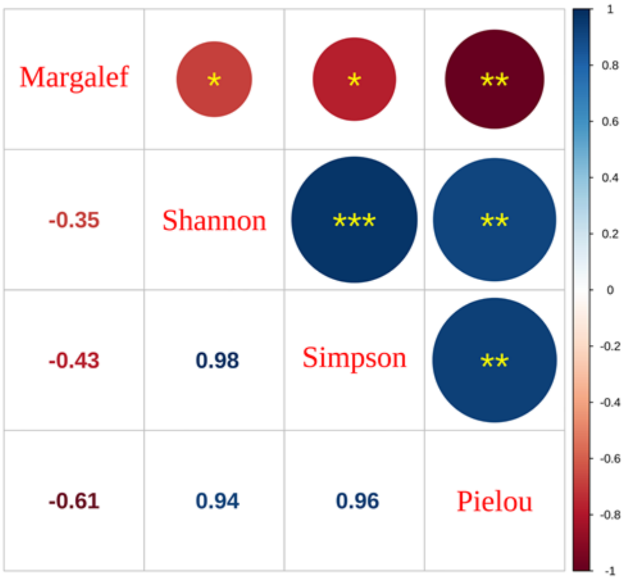

Figure S1. Correlation analysis of the species diversity index in the desert ecosystem. Note: Pearson correlation coefficients and their significance are given as ***: $P<0.001$; **: $P<0.01$; *: $P<0.05$. SW1 is the high moisture and salinity plot, and SW2 is the low moisture and salinity plot.
